# Road Toward a New Model of Care for Idiopathic Pulmonary Fibrosis in the Lazio Region

**DOI:** 10.3389/fmed.2022.861076

**Published:** 2022-06-09

**Authors:** Rossella Di Bidino, Paola Rogliani, Alfredo Sebastiani, Alberto Ricci, Francesco Varone, Giacomo Sgalla, Bruno Iovene, Teresa Bruni, Maria Chiara Flore, Michela D'Ascanio, Francesco Cavalli, Daniela Savi, Loreta Di Michele, Americo Cicchetti, Luca Richeldi

**Affiliations:** ^1^Graduate School of Health Economics and Management, Università Cattolica del Sacro Cuore (ALTEMS), Rome, Italy; ^2^Unit of Respiratory Medicine, Department of Experimental Medicine, Università di Roma “Tor Vergata, ” Rome, Italy; ^3^Department of Respiratory Diseases, San Camillo-Forlanini Hospital, Rome, Italy; ^4^Respiratory Unit, Sant'Andrea Hospital, Università di Roma Sapienza, Rome, Italy; ^5^Department of Pulmonary Medicine, Fondazione Policlinico Universitario Agostino Gemelli IRCCS, Rome, Italy; ^6^Department of Public Health and Infectious Diseases, Università di Roma Sapienza, Rome, Italy

**Keywords:** idiopathic pulmonary fibrosis, organizational analyses, real-world data analysis, care pathway analysis, therapeutic pathways

## Abstract

A timely, confirmed diagnosis of Idiopathic Pulmonary Fibrosis (IPF) has a significant impact on the evolution of the disease. The current model of care in the Lazio region (in Italy) was assessed on the basis of real-world data provided by the four reference centers responsible for diagnosing and treating IPF. The 5-year, population-based, retrospective longitudinal study provided the data that is at the basis of the current proposal for a new clinical and therapeutic pathway (DTCP) and has been shared with regional decision makers. A DTCP must be defined and based on four pillars: GPs, pulmonologists, IPF centers, and telemedicine. Each must play a role within a sort of hub-and-spoke model. IPF centers remain the hubs, while spokes are identified in trained GPs and pulmonologists.

## Introduction

Care pathways are able to impact on clinical outcomes. Translating clinical practice guideline recommendations into clinical processes is a critical aspect that should be constantly monitored. In the case of a progressive life-limiting disease, such as idiopathic pulmonary fibrosis (IPF), a timely and accurate diagnosis is essential. The patient pathway, through which a confirmed diagnosis of IPF is reached, could have a significant impact both in terms of access to available treatments and progression of the disease.

IPF is a specific and aggressive form of chronic fibrosing interstitial pneumonia. The diagnostic process is complex, and a definitive diagnosis must be based on clinical, laboratory, radiologic, and/or pathologic data. Late diagnoses could still occur due to difficulties in differentiating IPF vs. other interstitial lung diseases (ILD), and also due to a lack of knowledge of the disease (1, 2). Early recognition, diagnosis, and staging are crucial toward an improvement in outcomes.

In fact, access to antifibrotic therapies, such as nintedanib (3) and pirfenidone (4), is beneficial and considered in early stages of the disease. The Italian Medicines Agency has defined criteria for initiating treatment based on Forced Vital Capacity (FVC%), lung diffusing capacity for carbon monoxide (DLCO), and age (5, 6).

## Diagnostic and Therapeutic Care Pathways and Clinical Networks

In 2015, Italian Ministerial Decree n.70, dated 2 April 2015 (7) introduced the concept of Diagnostic-Therapeutic Care Pathways (in Italian: Percorsi Diagnostici Terapeutici Assistenziali, PDTA) as a tool for clinical governance, with the aim of mainly reorganizing chronic care. In addition, clinical networks were also promoted.

DTCPs cover both clinical and care aspects, aiming to harmonize provided assistance, guarantee continuum of care along the course of the disease, and improve accountability, while recognizing the role of different healthcare professionals. All involved stakeholders should participate in defining a disease specific DTCP. The definition of disease specific DTCP requires the involvement of all stakeholders and must be established on evidence-based medicine. Over time, specific performance indicators monitor each approved DTCP.

In the majority of cases, DTCPs are associated with hub-and-spoke clinical networks. Service is provided in a network form with an anchor establishment (hub), capable of offering a full array of services, and with affiliated centers (spokes), which offer a more limited number of services but that are still able to direct patients to more intensive services provided by the hubs.

## Regional IPF Scenario

Since 2018, four reference centers in the Lazio region (Italy) have been responsible for the diagnosis and treatment of IPF. These centers are: Pulmonology Unit at the Fondazione Policlinico Universitario Agostino Gemelli IRCCS, Fondazione PTV “Policlinico Tor Vergata,” Azienda Ospedaliera San Camillo Forlanini, and Azienda Ospedaliera-Universitaria Sant'Andrea (since 2018). All four IPF centers are located in Rome.

No regional clinical and therapeutic pathway (DTCP) has yet been defined for IPF. Nevertheless, only these centers with their multidisciplinary team (MDT) are able to confirm a diagnosis of IPF, perform high-resolution computed tomographies (HRCT), and prescribe antifibrotic therapies. Therefore, the lack of formalized referral criteria for IPF centers could have an impact on the possibility of accessing available treatments.

## Real Word Data on Regional Management of Idiopathic Pulmonary Fibrosis

A collaborative study was conducted by all four IPF reference centers. All of them defined and contributed to the research. A 5-year, population-based, retrospective longitudinal study was designed to assess how this hub-based model is effective in early diagnosis and therapeutic management.

The primary goal was to collect aggregate data on pre-diagnosis IPF patient pathways and patient characteristics. Evidence was then used to describe the current model of care. Then a SWOT analysis was conducted to identify strengths, weaknesses, opportunities, and threats associated with the current model of care. Results were shared and discussed during an in-person, multi-stakeholder meeting. Finally, opportunities for improvement were identified to promote and support the definition of a regional DTCP.

Similarly to other studies, we chose to conduct a survey that aimed at describing a real-world model of care for IPF (8–10).

A survey was then created to collect data on patients diagnosed with IPF through the four above mentioned IPF centers between 2014 and 2018. Previously published analyses for Italy refer to cases recorded in 2009 [in Lazio (11)] and in 2010 [in Lombardy (12)]. Our study not only took into consideration a more recent timeframe (from 2014 to 2018), but also a clinical scenario were patients could have access to specific IPF therapies. Pirfenidone was approved by AIFA in 2013 and nintedanib in 2016.

The survey was conducted from June to August 2019. Aggregated data was collected on demographic (age, gender, and province of residence), clinical (treatments before confirmed diagnosis), and organizational variables (i.e., personnel involved and healthcare services provided to patients).

The survey was only submitted to the four IPF centers participating in the study. Only IPF centers were involved, since all patients with suspected IPF had to refer to them for a confirmed diagnosis. Furthermore, the diagnostic criteria that were adopted for IPF were compared among centers at the beginning of the study and assessed as being comparable, also given the presence of a multidisciplinary team (MDT) in each of them. This guaranteed for homogeneity in case retrieval and comparativeness of data. The Pulmonology Unit of the Fondazione Policlinico Universitario Agostino Gemelli IRCCS collected and analyzed the data.

**Figure 1:**
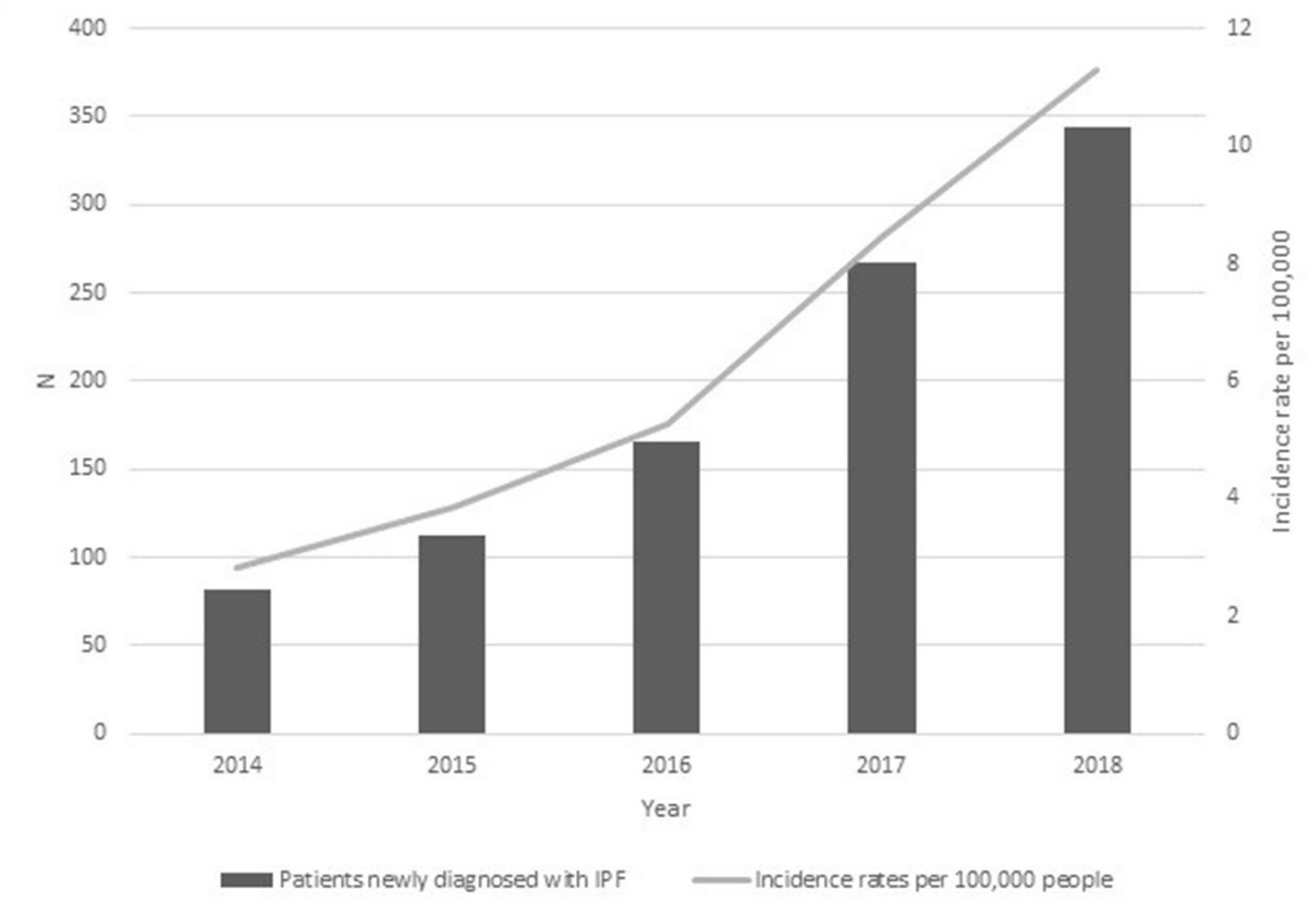
Patients with confirmed diagnosis of IPF in the Lazio region from 2014 to 2018. Absolute numbers and incidence rates per 100,000 people (>45 years of age).

Collected data showed that the number of patients with a newly confirmed diagnosis of IPF in the Lazio region increased from 82 in 2014 to 344 in 2018 (Table 1), with a significant growth in 2018 (+60.8% vs. 2017). This increase was confirmed, taking into consideration the incidence rate of the population of the Lazio region (Figure 1).

**Table 1:** Patients with confirmed diagnosis of IPF in the Lazio region from 2014 to 2018.

	**2014**	**2015**	**2016**	**2017**	**2018**
Patients newly diagnosed with IPF (*n*):	82	112	166	267	344
Annual change (%):		36.60%	48.20%	60.80%	28.80%
Incidence rates per 100,000 people:	2.82	3.84	5.28	8.44	11.30
**Access to IPF treatments**					
Patients eligible for treatment (*n*, %):	67 (88.2%)	90 (89.1%)	143 (93.5%)	237 (92.9%)	302 (94.1%)
Patients not eligible for treatment (*n*, %):	9 (11.8%)	11 (10.9%)	10 (6.5%)	18 (7.1%)	19 (5.9%)
**Reasons for lack of access to pharmacological treatments (** ***n*** ,%)*:					
FVC <50%	1 (7.1%)	0 (0%)	2 (8.7%)	1 (3.3%)	2 (4.8%)
DLCO <35% or DLCO <30%	5 (35.7%)	6 (30.0%)	2 (8.7%)	4 (13.3%)	4 (9.5%)
Age > 80 years	2 (14.3%)	3 (15.0%)	3 (13.0%)	2 (6.7%)	4 (9.5%)
Personal choice	5 (35.7%)	9 (45.0%)	16 (69.6%)	16 (53.3%)	25 (59.5%)
Other reason	1 (7.1%)	2 (10.0%)	0 (0%)	7 (23.3)	7 (16.7%)
**Patient characteristics**					
**Gender (** ***n*** **,%):**					
Male	62 (75.6%)	92 (82.1%)	116 (69.9%)	205 (76.8%)	255 (74.1%)
Female	20 (24.4%)	20 (17.9%)	41 (24.7%)	49 (18.4%)	87 (25.3%)
Missing data	0 (0%)	0 (0%)	9 (5.4%)	13 (4.9%)	2 (0.6%)
**Age (** ***n*** **, %):**					
<45	1 (1.2%)	1 (0.9%)	1 (0.6%)	1 (0.4%)	1 (0.3%)
45–49	1 (1.2%)	1 (0.9%)	0 (0%)	0 (0%)	5 (1.5%9
50–54	1 (1.2%)	1 (0.9%)	2 (1.2%)	5 (1.9%)	4 (1.2%)
55–59	5 (6.1%)	6 (5.4%)	7 (4.2%)	5 (1.9%)	6 (1.7%)
60–64	16 (19.5%)	16 (14.3%)	17 (10.2%)	14 (5.2%)	12 (3.5%)
65–69	12 (14.6%)	17 (15.2%)	24 (14.5%)	42 (15.7%)	56 (16.3%)
70–74	20 (24.4%)	29 (25.9%)	39 (23.5%)	71 (26.6%)	87 (25.3%)
75–79	22 (26.8%)	27 (24.1%)	40 (24.1%)	61 (22.8%)	97 (28.2%)
80–84	2 (2.4%)	11 (9.8%)	22 (13.3%)	40 (15.0%)	61 (17.7%)
85–89	1 (1.2%)	3 (2.7%)	4 (2.4%)	13 (4.9%)	14 (4.1%)
90–94	0 (0%)	0 (0%)	0 (0%)	1 (0.4%)	0 (0%)
>95	0 (0%)	0 (0%)	0 (0%)	0 (0%)	0 (0%)
Missing data	1 (1.2%)	0 (0%)	10 (6.0%)	14 (5.2%)	1 (0.3%)
**Incidence rates per 100,000 people per age group:**					
45–49	0.20	0.20	0.00	0.00	1.01
50–54	0.23	0.22	0.42	1.03	0.81
55–59	1.31	1.54	1.76	1.22	1.42
60–64	4.69	4.65	4.89	3.95	3.31
65–69	3.72	5.10	6.98	12.46	16.93
70–74	6.98	10.44	14.57	25.60	30.05
75–79	8.94	10.69	15.44	23.37	37.48
80–84	1.10	5.91	11.70	21.01	31.28
85–89	0.93	2.71	3.45	10.90	11.56
90–94	0.00	0.00	0.00	1.98	0.00
>95	0.00	0.00	0.00	0.00	0.00
**Geographical distribution of patients**					
**Residence (** ***n*** **,%):**					
Lazio region	75 (91.5%)	105 (93.8%)	148 (89.2%)	240 (89.9%)	313 (91.0%)
Outside Lazio region	7 (8.5%)	7 (6.3%)	8 (4.8%)	13 (4.9%)	29 (8.4%)
Missing data	0 (0%)	0 (0%)	10 (6.0%)	14 (5.2%)	2 (0.6%)
**Province of residence (in Lazio region) (** ***n*** **):**					
Roma	61 (81.3%)	80 (76.2%)	113 (76.4%)	195 (81.3%)	250 (79.9%)
Viterbo	3 (4.0%)	2 (1.9%)	4 (2.7%)	9 (3.8%)	9 (2.9%)
Frosinone	6 (8.0%)	9 (8.6%)	14 (9.5%)	17 (7.1%)	27 (8.6%)
Rieti	1 (1.3%)	7 (6.7%)	6 (4.1%)	5 (2.1%)	8 (2.6%)
Latina	4 (5.3%)	7 (6.7%)	11 (7.4%)	14 (5.8%)	19 (6.1%)
**Incidence rates per 100,000 people per province of residence:**					
Roma	2.90	3.74	5.20	8.84	11.17
Viterbo	1.82	1.20	2.38	5.31	5.26
Frosinone	2.44	3.62	5.58	6.72	10.60
Rieti	1.19	8.26	7.02	5.83	9.28
Latina	1.49	2.56	3.96	4.96	6.63
**Clinical pathways, pre-IPF diagnosis**					
**Previous visits with (** ***n*** ,%)*of treatable patients ( ***n*** **):**					
GP**	14 (16.1%)	15 (13.8%)	20 (13.4%)	31 (13.0 %)	49 (15.6%)
Pulmonologist	62 (71.3%)	63 (57.8%)	91 (61.4%)	154 (64.7%)	205 (65.1%)
Radiologist	0 (0%)	2 (1.8%)	2 (1.3%)	4 (1.7%)	2 (0.6%)
Rheumatologist	0 (0%)	0 (0%)	1 (0.7%)	5 (2.1%)	7 (2.2%)
Cardiologist	0 (0%)	0 (0%)	1 (0.7%)	3 (1.3%)	4 (1.3%)
Other IPF center	6 (6.9%)	17 (15.6%)	18 (12.1%)	11 (4.6%)	13 (4.1%)
Personal choice/decision	5 (5.7%)	9 (8.3%)	12 (8.1%)	25 (10.5%)	27 (8.6%)
Private healthcare organization	0 (0%)	3 (2.8%)	3 (2.0%)	4 (1.7%)	6 (1.9%)
Other (specify)	0 (0%)	0 (0%)	1 (0.7%)	1 (0.4%)	2 (0.6%)

* *Multiple choices were possible. % calculated on total number of responses*.

** *GP, General Practitioner*.

During the study period, the number of patients eligible for treatment increased both in absolute (from 67 to 302) and relative values (from 88.2 to 94.1% of annual incidence cases). This means patients received a confirmed IPF diagnosis in the early stages of the disease. For this study, we chose to adopt the same criteria as AIFA (Italian Medicines Agency) to identify reasons of ineligibility for treatment. In particular, a significant reduction in the proportion of ineligible patients due to DLCO levels emerged (35.4 to 9.5% – Table 1).

Our data confirm that IPF mainly affects males (74.1% in 2018) and that incidence of IPF increases with age. Diagnosis before age 45 was rare. The majority of cases were in the age range between 70 and 79 years. Patients > 80 years of age increased in relative terms from 3.7 to 21.8% during the study period. The incidence rate was 2.82 per 100,000 residents (age ≥ 45 years) in 2014 and reached 11.30 per 100,000 residents in 2018. Table 1 reports the change in incidence rate per age group per 100,000 residents from 2014 to 2018. Previous regional studies (11) estimated an annual incidence of IPF in a range from 0.4 per 100,000 (CI 95%, 0.3–0.4) in the 18–34 age group to 28.1 per 100,000 (CI 95%, 27.129.2) in individuals 75 years of age or older. The discrepancies are due not only to different timeframes (2005–2009), data sources, and criteria used to define a case of IPF, but mainly to the evolution of the age structure of the population. The findings are in line with previous reports (13, 14), where a rising incidence of the disease over time had already emerged. The increase in the number of IPF cases (and incidence rates) could be explained also by a better awareness that health professionals have of the disease at different levels of the Regional Healthcare System as results of specific educational initiatives.

Almost all cases of confirmed diagnosis resided in the Lazio region (91% in 2018) and in the province of Rome (79.9% in 2018). Rates per 100,000 residents confirmed a major incidence in the province of Rome (11.17 per 100,000 in 2018), even if the difference compared to Frosinone (10.60 per 100,000), and Rieti (9.28 per 100,000) was small. The increasing trend is confirmed by the incidence rate at a provincial level.

In addition, most of the treatable patients had previously visited with a pulmonologist (67.9% in 2018) and/or a GP (16.2% in 2018). Transfer between IPF centers appeared low (only 4.3% of patients moved from one center to another in 2018).

## IPF Center Organization

A multidisciplinary team was already active in all of the IPF centers. The structure of these teams varied slightly. Six additional pulmonologist (in three centers) and three nurses were the major changes applied, in order to respond to the significant increase in the patient population. Overall, eleven additional staff members were reported.

The average number of open days increased from 1.3 to 3.7% per week. None of the IPF dedicated outpatient services was open on Saturday. Since 2017, two pulmonology outpatient services have remained open 5 days per week.

The healthcare services provided were quite homogenous among the three active centers during the entire study period. Two (out of three) IPF centers were able to offer echocardiograms. While lung biopsies were always available, only one center was able to provide respiratory physiotherapy, mobility rehabilitation, and psychological consultations.

## Network for IPF on Site

As demonstrated by the data collected (Tables 1, 2), a *de facto* network for diagnosing and managing IPF patients has been operative in the Lazio region. The four IPF centers can count on multidisciplinary teams for patient management. There is, however, room for improvement because, in the absence of an official and shared definition of a regional DTCP, the process the patient must follow is quite erratic when attempting to reach a qualified center. In addition, all four IPF centers are located in Rome, while a not so irrelevant number of patients live in other areas of the region. To prevent this additional burden on patients, several activities (i.e., disease monitoring) could be delegated to spoke centers. During the study period, a key potential role emerged that had already been played by pulmonologists and GPs.

**Table 2:** IPF Centers: available resources and services provided.

	**2014**	**2015**	**2016**	**2017**	**2018**
**Personnel** (total number in 3 centers)					
Pulmonologists	8	10	11	12	14
Radiologist	6	6	5	6	7
Pathologist	3	3	3	3	3
Nursing staff	4	4	4	4	7
Multidisciplinary team coordinator	3	3	3	3	3
Administrative staff	2	2	2	2	3
**Outpatient activity**					
Number of open days per week (mean)	1.3	2.7	2.7	2.7	3.7
Number of open hours per weekday (mean):					
**IPF dedicated outpatient service:**					
Monday	2.3	4.0	5.5	4.7	4.7
Tuesday	0.0	2.5	2.5	2.0	2.0
Wednesday	3.0	2.5	2.5	2.5	2.0
Thursday	0.0	2.5	2.5	2.5	2.0
Friday	3.0	5.5	2.5	2.5	2.0
Saturday	0.0	0.0	0.0	0.0	0.0
Pulmonology outpatient service:					
Monday	3.7	3.7	3.7	3.7	3.7
Tuesday	5.5	5.5	5.5	5.7	5.7
Wednesday	5.5	5.5	5.5	5.5	5.7
Thursday	5.5	5.5	5.5	5.5	5.7
Friday	2.5	2.5	5.5	5.5	5.7
Saturday	0.0	0.0	0.0	2.5	3.7
**Healthcare services (number of centers):**					
Clinical/disease monitoring	3	3	3	3	3
Spirometry	3	3	3	3	3
Spirometry + DLCO	3	3	3	3	3
Blood gas analysis or saturation	3	3	3	3	3
6MWT test	3	3	3	3	3
Echocardiogram	2	2	2	2	2
Thoracic HRCT	3	3	3	3	3
Polysomnography/Saturimetry	3	3	3	3	3
Oxygen therapy	3	3	3	3	3
**Clinical examinations/procedures (number of centers):**					
Respiratory physiotherapy	1	1	1	1	1
Mobility rehabilitation	1	1	1	1	1
BAL/TBB Broncoscopy	3	3	3	3	3
Lung biopsy	3	3	3	3	3
Psychological consultation	1	1	1	1	2

*DLCO, Carbon monoxide diffusing capacity; 6MWT, Six minute walking test; BAL, Bronchoalveolar Lavage; TBB, Transbronchial Biopsy*.

## Areas of Improvement

Opportunities for improvement were identified in the definition of criteria that justify the decisions of GPs to refer a patient with suspected symptoms to a specialized pulmonologist and then to an IPF center. In addition, the selection and definition of indicators which can adequately monitor the key phases of the diagnostic and therapeutic pathway were proposed. Furthermore, multidisciplinary teams should be formalized both in terms of structure and size (number of health professionals) in relation to workload (i.e., number of diagnosed and monitored patients).

Moreover, the aging of the population, as well as the evolution of natural disease history (13–15), are aspects to consider when planning an appropriate regional health policy. Finally, training of all health professionals involved in the IPF clinical pathway is one of priorities that should be addressed.

## Involvement of Regional Decision Makers

Our study demonstrates the potentialities of a collaborative study that aims at collecting real-world data, in order to prove the need to optimize the current model of care. The dissemination phase was accurately planned. Results were shared with regional decision makers (16). An in-person meeting was organized to stimulate stakeholder engagement (17) in December 2019. Patient representatives, providers, policy makers, and researchers were invited.

**Figure 2:**
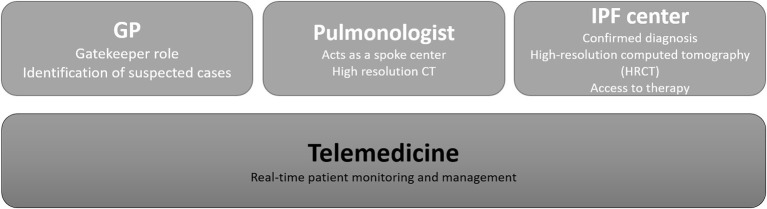
Pillars of a new model of care for IPF in the Lazio region.

During the multi-stakeholder meeting, results were further discussed in terms of patient population evolution, workload, and available resources for IPF centers. The SWOT analysis (Table 3) was the first step toward identifying the three pillars for a future regional DTCP for IPF. Real-world data demonstrated the crucial role already played by GPs, pulmonologists, and qualified centers, and are proposed as the basic pillars for the new model of care (Figure 2). Communication among those involved must be improved, in order to reach an effective pathway that can identify suspected cases, refer them to the most appropriate node, and confirm diagnosis in a clinically acceptable timeframe. Improving communication would then also improve quality of care. Strong connections among these three nodes would allow to adequately monitor patients and anticipate or rapidly manage exacerbations.

**Table 3:** SWOT analysis of the IPF model of care from 2015 to 2018.

**Strengths**	**Weaknesses**
A network for diagnosing and managing IPF patients is already operative. Four IPF qualified centers are present. Multidisciplinary teams are already active.	Lack of an official definition at a regional level for the diagnostic and therapeutic pathway. Patient's first contact with an IPF center should be optimized. All IPF centers are located in Rome. A redefinition of the geographical distribution of activities (diagnosis, monitoring, and routine care) should be considered.
**Opportunities**	**Threats**
Definition of criteria and objective indicators for monitoring key phases of the diagnostic and therapeutic pathway. Definition of criteria for admitting a patient to an IPF center. Definition of the structure of a multidisciplinary team (in terms of qualification and number of healthcare professionals, in relation to volume of activity). Definition of criteria in monitoring IPF activities, in relation to volume of patients managed.	Evolution of the natural history of IPF. Aging of the population. Training and communicating activities for GPs and pulmonologists, in order to increase awareness and knowledge of IPF.

Telemedicine is the fourth pillar. This is essential in allowing for and maintaining communication among GPs, pulmonologists, IPF centers, and patients. Positive experiences regarding the use of e-health solutions for IPF patients have already been reported (18–20). We propose to integrate them with specific alerts for each pillar and also criteria that justify the involvement of other solutions. GPs could be responsible for routine monitoring while, when exacerbation is expected or conditions worsen, pulmonologists must be involved. Following clinical examinations, IPF centers could more appropriately intervene and decide upon further testing, changes in therapy, and monitoring. To reach this level of development, different stakeholders should participate in its definition and implementation.

The positive impact of telemedicine on the management of IPF cases has been already demonstrated both in studies conducted during the pandemic and in trials. It has been demonstrated the ability of telemedicine to both reduce exacerbations and hospitalizations (21). In addition, it allowed more individually tailored medication adjustments (22). What we propose is a more systematic use of telemedicine not only in the monitoring of patients, but also in the management of the whole DTCP.

## Actionable Recommendations

A regional clinical and therapeutic pathway (DTCP) is requested for defining and managing the multi-pillar system. Communication and training should be guaranteed, and an integrated telemedicine solution should be defined and managed at a regional level. Coordination in the diffusion of telemedicine is crucial. For instance, single initiatives at healthcare provider level can have only a limited impact on the patient pathway. An integrated platform or system is the only way to respond to both decision makers and the needs of patients. In addition, a coordinated diffusion of telemedicine has positive externalities on clinical research, because it will allow to have a unique real word database based on uniform criteria of data collection for IPF. As a consequence, it will be possible both to monitor real time the evolution of the disease, and to have real world evidence (RWE) on the effectiveness of available treatments and on the impact of DPCP.

In Italy, two regions (Lombardia, Veneto) have already defined a DTCP for IPF. Both are transparent regarding the structure of multidisciplinary teams. Only the Lombardia region defined the role of different clinicians in relation to the phases of the pathway (clinician examination, diagnosis, treatment definition, and communication) (23), while the Veneto region formulated a list of indicators that monitor activities in IPF centers (24).

The proposal elaborated (and described here) for the Lazio region will represent the first case of a regional DTCP explicitly involving various health professionals, even external to IPF centers, and will give relevance to digital solutions. It can be interpreted as a hub-and-spoke model. IPF centers remain the hubs, while spokes are identified in trained GPs and pulmonologists.

During the meeting, all stakeholders agreed on the need for a better defined clinical pathway. Policy makers supported the proposal to involve other players, external to the IPF centers, in order to guarantee a continuity in adequate care. Patient representatives positively reacted to the multi-stakeholder approach suggested for developing a new DTCP. In addition, the implementation of telemedicine was welcomed. Finally, an increase in awareness and competencies in terms of IPF diagnosis emerged as crucial points for an efficient new DTCP.

In the end, both regional and hospital-specific databases can contribute to a more complete analysis of patient pathways. A larger collaborative study would be a relevant further development.

The approach proposed for IFP could guide the definition or refinement of DTCP of other interstitial lung diseases (ILSs). In particular, the same four pillars approach could be quite similar in pre-the diagnosis phase for ILSs that share common symptoms and required the involvement of the same health professionals. Then, the role of telemedicine can be adapted to the specific disease pathway.

## Conclusions

A 5-year, population-level, retrospective longitudinal study was not only able to demonstrate a significant increase in the ability to detect IPF cases, but also to identify the key players of the current model of care based on four qualified IPF centers. A SWOT analysis allowed to elaborate a proposal that was introduced and discussed during a multi-stakeholder meeting in which patient representatives and also regional decision makers participated. The proposal of a three-pillar system (GPs, pulmonologists, and IPF centers) supported by telemedicine was well accepted. This would represent the first case of a regional DTCP explicitly involving different health professionals, even external to IPF centers, and would give relevance to digital solutions. To further elaborate and implement this, a multi-stakeholder approach should be maintained.

## Data Availability Statement

The original contributions presented in the study are included in the article/supplementary material, further inquiries can be directed to the corresponding author.

## Ethics Statement

The study was communicated to the Ethical Commitee of each IPF center. Written informed consent was not required to participate in this study in accordance with the national legislation and the institutional requirements.

## Author Contributions

All authors listed have made a substantial, direct, and intellectual contribution to the work and approved it for publication.

## Funding

The authors received unrestricted financial support by Boehringer Ingelheim.

## Conflict of Interest

All authors has received a research grants from ALTEMS to participate to the study. For other studies, LR has received consulting fees from Boehringer Ingelheim, Roche, FibroGen, Celgene, RespiVant, Nitto, Bristol Myers Squibb, Pliant Therapeutics, Veracyte, CSL Behring, Novartis, and speaker honorarium from Boehringer Ingelheim, Roche, Zambon, CSL Behring and participated in Data Safety Monitoring Board or Advisory Boards for Boehringer Ingelheim, Roche, FibroGen, Veracyte. Departement of Experimental Medicine - Respiratory Medicine (Università di Roma “Tor Vergata”) was funded by Almirall, Boehringer Ingelheim, Chiesi Farmaceutici, Novartis, and Zambon. PR participated as a lecturer in scientific meetings and courses under the sponsorship of Almirall, AstraZeneca, Biofutura, Boehringer Ingelheim, Chiesi Farmaceutici, GlaxoSmithKline, Menarini Group, Mundipharma, and Novartis and as an advisor in scientific meetings and courses under the sponsorship of Almirall, AstraZeneca, Biofutura, Boehringer Ingelheim, Chiesi Farmaceutici, GlaxoSmithKline, Menarini Group, Mundipharma, and Novartis. AS has received consulting fee from Boehringer Ingelheim, Roche and participated in Data Safety Monitoring Board or Advisory Boards for Roche. Then received support for attending meetings and/or travel from Roche. FV has received a speaker honorarium from from Boehringer Ingelheim, Roche. The remaining authors declare that the research was conducted in the absence of any commercial or financial relationships that could be construed as a potential conflict of interest.

## Publisher's Note

All claims expressed in this article are solely those of the authors and do not necessarily represent those of their affiliated organizations, or those of the publisher, the editors and the reviewers. Any product that may be evaluated in this article, or claim that may be made by its manufacturer, is not guaranteed or endorsed by the publisher.
